# Suppressive actions of eicosapentaenoic acid on lipid droplet formation in 3T3-L1 adipocytes

**DOI:** 10.1186/1476-511X-9-57

**Published:** 2010-06-04

**Authors:** Elizabeth Manickam, Andrew J Sinclair, David Cameron-Smith

**Affiliations:** 1 Molecular Nutrition Unit, School of Exercise and Nutrition Sciences, Faculty of Health, Medicine, Nursing and Behavioural Sciences, Deakin University, Melbourne, Victoria 3125, Australia; 2 School of Medicine, Faculty of Health, Medicine, Nursing and Behavioural Sciences, Deakin University, Geelong, Victoria 3125, Australia

## Abstract

**Background:**

Lipid droplet (LD) formation and size regulation reflects both lipid influx and efflux, and is central in the regulation of adipocyte metabolism, including adipokine secretion. The length and degree of dietary fatty acid (FA) unsaturation is implicated in LD formation and regulation in adipocytes. The aims of this study were to establish the impact of eicosapentaenoic acid (EPA; C20:5n-3) in comparison to SFA (STA; stearic acid, C18:0) and MUFA (OLA; oleic acid, C18:1n-9) on 3T3-L1 adipocyte LD formation, regulation of genes central to LD function and adipokine responsiveness. Cells were supplemented with 100 μM FA during 7-day differentiation.

**Results:**

EPA markedly reduced LD size and total lipid accumulation, suppressing PPARγ, Cidea and D9D/SCD1 genes, distinct from other treatments. These changes were independent of alterations of lipolytic genes, as both EPA and STA similarly elevated LPL and HSL gene expressions. In response to acute lipopolysaccharide exposure, EPA-differentiated adipocytes had distinct improvement in inflammatory response shown by reduction in monocyte chemoattractant protein-1 and interleukin-6 and elevation in adiponectin and leptin gene expressions.

**Conclusions:**

This study demonstrates that EPA differentially modulates adipogenesis and lipid accumulation to suppress LD formation and size. This may be due to suppressed gene expression of key proteins closely associated with LD function. Further analysis is required to determine if EPA exerts a similar influence on LD formation and regulation *in-vivo*.

## Background

Adipose tissue is a complex dynamic tissue facilitating energy storage, which also exerts considerable influence on whole body metabolic function through secreted hormones and adipokines [1]. Importantly, the size of adipocytes is an important determinant of the profile of the adipokines secreted, with large adipocytes predominately releasing pro-inflammatory factors such as monocyte chemoattractant protein-1 (MCP-1) and interleukin-6 (IL-6), and reduced anti-inflammatory adipokines including leptin and adiponectin [2-4]. In adipocytes, triacylglycerols (TAG) are sequestered within lipid droplets (LDs), with over 95% of each mature adipocyte is composed of TAG. LD formation is the major pathway regulating adipocyte size [5].

LDs are far from inert vesicles, and are composed of a central core of neutral lipids surrounded by a phospholipid monolayer that occurs in close association with a complex array of proteins [6]. Crucial in the genesis of LDs in adipocyte differentiation from precursor fibroblasts is the developmental program initiated and regulated by key adipogenic transcription factors such as peroxisome proliferator-activated receptor γ (PPARγ) [7].

The maintenance of LDs in mature adipocytes is regulated in part both by the TAG influx dictated by enzymes including lipoprotein lipase (LPL), and the predominant enzymes regulating TAG efflux, including adipose triglyceride lipase (ATGL, also known as desnutrin and Pnpla2) and hormone sensitive lipase (HSL, also known as *Lipe*) [8-10]. The activity of these efflux lipolytic enzymes is orchestrated by protein-protein interactions with Perilipin A, a lipid droplet scaffold protein [11]. Alterations in HSL expression has limited impact on LD size, however, alterations in ATGL activity profoundly influence LD size, independent from Perilipin A activity [12]. However, Perilipin A null mice have impaired LD formation, demonstrating that Perilipin A also uniquely influences LD function [13]. Whilst the abundance of ATGL and Perilipin A demonstrate the importance of lipolytic control in regulating LD size, RNAi screening unexpectedly highlighted a close association and importance of the cell death-inducing DFF45-like effector (CIDE) domain containing protein (Cidea) with LD size [14]. The mice Cidea (homologous to humans CIDEA), previously described as a mitochondria-associating protein, associates with LDs and negatively regulates lipolysis, promoting increased LD size. Both Perilipin A and Cidea are transcriptionally regulated by PPARγ, demonstrating the importance of this pathway in the regulation of LD formation and size [15,16].

Depending on the length and degree of unsaturation, FAs have been predicted to influence PPARγ-regulated gene expression and subsequent LD formation during adipocyte differentiation [17,18]. Recently, adipocyte size has been noted to be influenced by the FA composition in the LDs that is regulated by delta-9 desaturase/stearoyl Co-A desaturase 1 (D9D/SCD1) gene [19] that is PPARγ-dependent [20]. Potent in the regulation of PPARγ is the long chain n-3 PUFA, eicosapentaeneoic acid (EPA). Diets enriched in EPA lower adipose tissue mass and suppress obesity development in rats [21]. Within adipocytes EPA is known to induce expression of genes for mitochondrial biogenesis and oxidative metabolism, increasing the catabolism of lipids [22,23]. Furthermore, EPA has been previously reported to attenuate pro-inflammation in favour of anti-inflammatory adipokines [23]. Yet in spite of this, the impact of EPA on the molecular mechanisms governing the key pathways dictating adipocyte size and the biogenesis of intracellular LDs has yet to be analysed.

In this study, the differential effects of 7-day period of EPA (C20:5n-3) compared with stearic (STA, C18:0) and oleic acids (OLA, C18:1n-9) in differentiating 3T3-L1 adipocytes was assessed on the basis of the molecular factors critical in governing LD biogenesis. The ability of the resultant adipocytes to modulate the expression of pro-inflammatory, compared to anti-inflammatory adipokines upon bacterial endotoxin lipopolysaccharide (LPS) challenge were also evaluated. It was hypothesised that EPA would suppress LD formation and enhance the anti-inflammatory adipokines in response to LPS challenge.

## Results

### The effect of FA on LD formation in 3T3-L1 adipocytes

**Figure 1:**
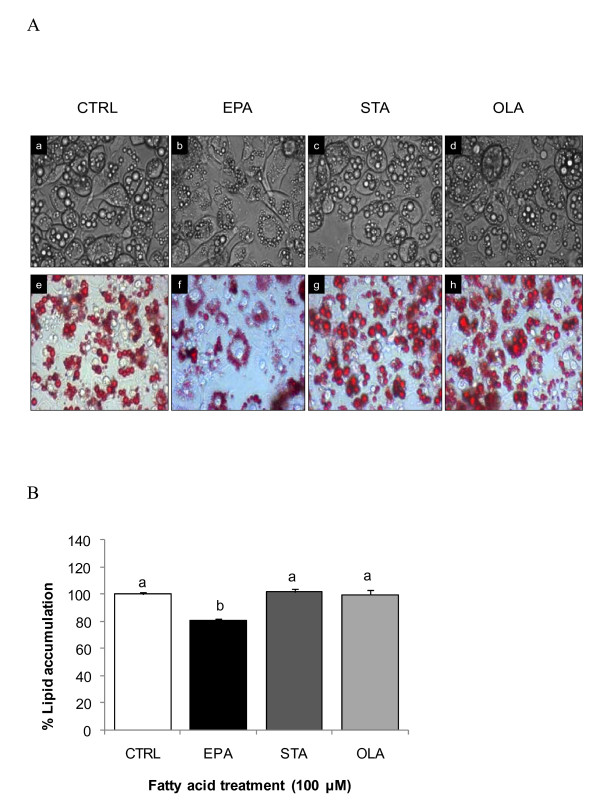
**Effects of FAs on adipocyte morphology and lipid accumulation**. The 3T3-L1 pre-adipocytes were incubated in growth medium supplemented with adipogenic factors (520 μM IBMX, 200 nM Dex and 2 μg/mL insulin). FAs (100 μM) of EPA, STA or OLA were included throughout 7 days of adipocyte differentiation. A) Cell morphology of differentiated adipocytes analysed with phase contrast microscopy before (a - d) and after (e - h) Oil Red O stain. B) Stained lipid fraction was measured spectrophotometrically on Day 8 at A_520 nm_. Values were calculated as percent lipid content versus vehicle control cells treated with 2% BSA + 100% ethanol alone (CTRL) and are expressed as means ± SEM (n = 6). Values with different superscript letters are significantly different at *P *< 0.05 by Tukey's post hoc test.

A representative image of LD size and number of 3T3-L1 adipocytes which underwent differentiation in the presence of differing FA is shown in Figure 1A. Adipocytes differentiated in the absence of added FA (CTRL), or with STA and OLA after 7 days resulted in larger LDs than that observed with EPA treatment. The smaller LD observed with EPA treatment reduced total lipid accumulation by 20% (*P *< 0.05), as measured using Oil Red O staining, but no difference was observed in other groups compared with CTRL (Figure 1B).

### Gene expression of adipogenic and LD factors

**Figure 2:**
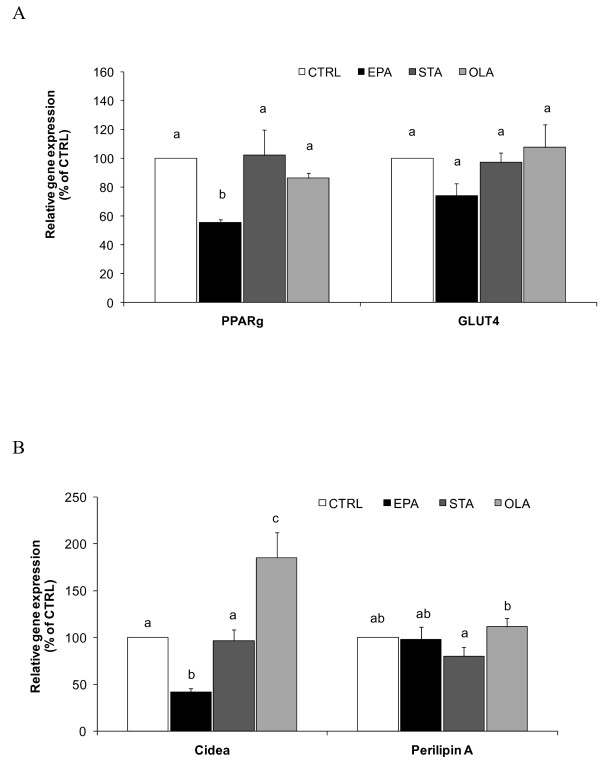
**Effects of FAs on mRNA expression of (A) adipogenesis; PPARγ and GLUT4, and (B) LD size; Cidea and Perilipin A**. Cells were differentiated in the presence of 100 μM FA or vehicle control. EPA, in contrast to STA and OLA, resulted in a significant inhibition of Cidea and PPARγ gene expressions but not Perilipin A or GLUT4. All values are means ± SEM of percent gene expression relative to CTRL, where cDNA levels were normalised by cyclophilin levels (n = 4). Values with different superscript letters are significantly different at *P *< 0.05 by Tukey's post hoc test.

The impact of EPA, in comparison to STA and OLA, on PPARγ and GLUT4, is shown in Figure 2A. EPA treatment during adipocyte differentiation decreased PPARγ by nearly 50% compared with STA or OLA. GLUT4 gene expression was unaltered by all treatments. Furthermore, genes central to LD formation (Cidea and Perilipin A) were also measured (Figure 2B). EPA significantly decreased Cidea gene expression by 50% (*P *< 0.05) compared with CTRL or STA. Comparatively, OLA increased Cidea gene expression. Perilipin A gene expression was unaltered from CTRL in all groups.

### Gene expression of lipolytic enzymes

**Figure 3:**
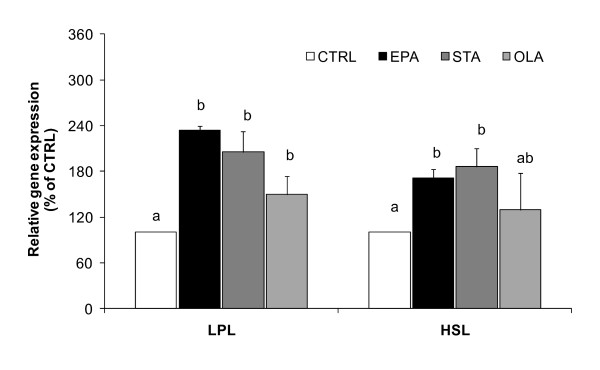
**Effects of FAs on mRNA expression of lipolytic enzymes, LPL and HSL**. Adipocytes were differentiated in the presence of 100 μM FA or vehicle control. All FA treatments, EPA, STA and OLA, increased gene expression of LPL and HSL, compared with vehicle control (CTRL). All values are means ± SEM of percent gene expression relative to CTRL, where cDNA levels were normalised by cyclophilin levels (n = 4). Values with different superscript letters are significantly different at *P *< 0.05 by Tukey's post hoc test.

As shown in Figure 3, there was a significant increased expression of LPL and HSL genes in adipocytes differentiated with all types of FAs relative to CTRL (*P *< 0.05). More than 2-fold increase in LPL gene expression was noted in adipocytes differentiated in the presence of EPA and STA, compared with CTRL. The expression of HSL gene was also significantly elevated by both EPA and STA, compared with CTRL (*P *< 0.05).

### Gene expression of D9D/SCD1

**Figure 4:**
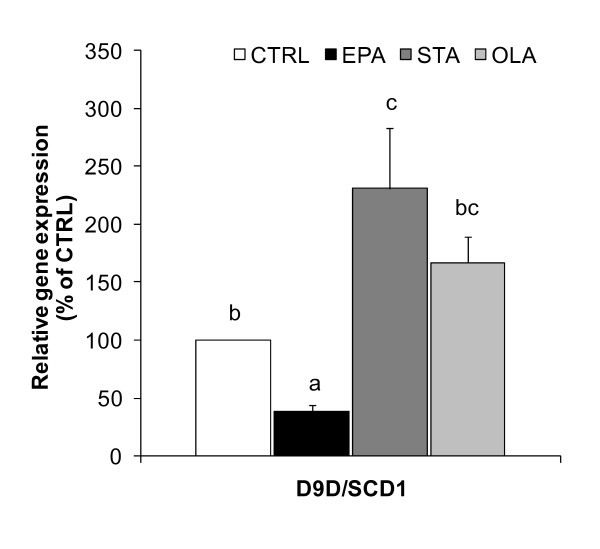
**Effects of FAs on mRNA expression of D9D/SCD1**. Cells were differentiated in the presence of 100 μM FA or vehicle control. EPA, in contrast to STA and OLA, resulted in a significant inhibition of D9D/SCD1 gene expression. All values are means ± SEM of percent gene expression relative to CTRL, where cDNA levels were normalised by cyclophilin levels (n = 4). Values with different superscript letters are significantly different at *P *< 0.05 by Tukey's post hoc test.

A significant elevation of D9D/SCD1 gene expression was evident in adipocyte differentiated with STA (131%) relative to CTRL (*P *< 0.05) (Figure 4). When EPA was introduced, the adipocytes expressed 62% less D9D/SCD1 gene expression compared with CTRL (*P *< 0.05).

### Gene expression of inflammatory adipokines upon LPS challenge

The changes in the inflammatory adipokines upon LPS challenge in differentiated-adipocytes are shown in Table 1. The elevation of MCP-1 gene expression after LPS treatment was significantly lower in EPA-differentiated adipocytes, compared with CTRL or STA (5.6-fold *vs *> 13-fold) (*P *< 0.05). Similarly, the expression of IL-6 gene was 4.1-fold less after EPA treatment compared with STA. Alternatively, the gene expressions of adiponectin (2.2-fold *vs *1.1-fold) and leptin (2.8-fold *vs *1.2-fold) were significantly elevated upon LPS challenge in EPA compared with STA treatment.

**Table 1:** Gene expression of inflammatory adipokines upon LPS challenge in adipocytes after differentiation with EPA, STA, OLA or vehicle control

Gene expression of adipokines (fold change/unstimulated)	FA treatment (100 μM)			
	
	CTRL	EPA	STA	OLA
MCP-1	13.9 ± 3.1^a^	5.6 ± 0.8^b^	13.4 ± 3.3^a^	6.6 ± 1.8^ab^
IL-6	7.2 ± 0.8^ab^	5.5 ± 0.8^b^	9.6 ± 0.9^a^	5.2 ± 0.8^b^
Adiponectin	1.4 ± 0.1^ab^	2.0 ± 0.4^b^	1.1 ± 0.2^a^	1.3 ± 0.2^ab^
Leptin	0.9 ± 0.2^a^	2.8 ± 1.0^b^	1.2 ± 0.2^a^	1.0 ± 0.3^a^

Values are means ± SEM of n = 4, with different superscript letters are significantly different at *P *< 0.05 by Tukey's post hoc test.

## Discussion

LDs are important organelles, not only for cellular energy storage, but emerging research demonstrates a central role in intracellular signalling and adipokine regulation. Thus, the formation and regulation of LDs may contribute to the metabolic disease progression towards type 2 diabetes and cardiovascular disease evident in obesity. FAs are important in both providing the substrate for LD formation, and may also be central in regulating LD formation and size. Previously, long chain n-3 PUFA have been reported to suppress LD formation [24,25], although comparison to other FA and putative mechanisms is lacking. The present study demonstrated a marked suppression of LD formation in 3T3-L1 adipocytes maintained in the presence of EPA, when compared to either a SFA or MUFA. Of the mechanisms governing LD formation and regulation, EPA suppressed PPARγ, Cidea and D9D/SCD1 gene expressions, while maintaining the expression of lipolytic genes, including LPL and HSL. A key regulator of adipogenesis is PPARγ, and FA sensitive transcriptional regulator of many thousands of adipocyte-specific genes [7].

Specifically, both n-6 and n-3 PUFA species directly influence PPAR activity [23]. Although known as an acute agonist and regulator of the PPARγ gene [26], chronic EPA exposure was shown to suppress this gene expression. Many of the subsequent genes analysed in the present study are PPAR-dependent, yet the responsiveness was varied, suggesting alteration of PPAR activity was not the sole regulating factor suppressing LD formation in the presence of EPA.

Cidea and Perilipin A are proteins highly localised to LDs of adipose tissue that are important for FA esterification to TAG and lipid mobilisation [14,27]. Cidea and Perilipin A are known to be closely regulated by PPARγ [16]. Cidea acts as a shield for fat storage that drives lipid accumulation even in cells that rarely store fat [28]. Similarly, Cidea expression is abundantly upregulated with high SFA feeding [14]. Conversely, animals or humans with Cidea deficiency display lean phenotypes with resistance to diet-induced obesity [29]. In this study, Cidea gene expression was downregulated in adipocytes treated with EPA but not after STA or OLA treatments.

Perilipins are a class of proteins closely associated with LD formation in adipocytes [30], yet the critical relevance of this gene in the regulation of lipid mobilisation upon nutritional challenges in adipocytes is uncertain. An elevated Perilipin A level in obese humans [31] is inconsistent with decreased diet-induced obesity in Perilipin A-overexpressed mice [32]. In this study, we found that EPA significantly affected Cidea but not Perilipin A. The substantial reduction in Cidea gene expression by EPA, but not by STA or OLA, is consistent with the decreased LD size and PPARγ, suggesting that Cidea (along with PPARγ) is the prime target for EPA in adipocytes, not Perilipin A.

Another possible mechanism governing LD formation is linked to D9D/SCD1. Desaturases are other enzymes that are greatly affected by the degree of unsaturation of FAs. D9D/SCD1 is one particular desaturase that has been highly discussed in terms of SFA-rich diet-induced obesity. D9D/SCD1 is a key regulatory enzyme required to convert SFA to MUFA that participate in lipid metabolism in adipocytes [33,34]. The importance of D9D/SCD1 expression in the formation of larger adipocytes has also been recently noted [19]. Contrary to the upregulation shown by SFA and MUFA, dietary PUFA (arachidonic and α-linolenic acids) consistently decreased the expression of D9D/SCD1 in liver and adipose tissue of obese models [35,36]. Similar to the shorter chain n-3 PUFA α-linolenic acids, our study is the first to confirm a distinct downregulation of D9D/SCD1 in adipocytes after longer chain n-3 PUFA EPA treatment, in contrast to the upregulation shown by STA and OLA.

**Figure 5:**
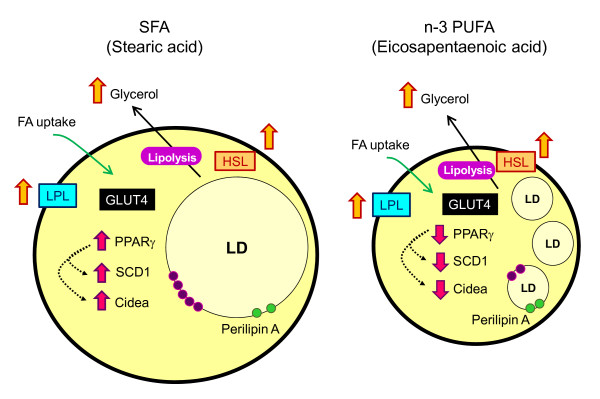
**Possible mechanism involved in FA-regulated LD formation in adipocytes**. Both SFA (stearic acid) and n-3 PUFA (eicosapentaenoic acid) have similar magnitude of effects towards lipolysis controlled by LPL and HSL. In contrast to upregulation shown by SFA, n-3 PUFA decreased PPARγ level, which may be responsible for the reduction of D9D/SCD1 and Cidea expressions, leading to smaller LDs. The levels of GLUT4 and Perilipin A remain unchanged upon FA treatment.

Like many other adipocyte-specific genes, D9D/SCD1 is also upregulated by PPARγ during adipogenesis, while PPARγ expression is not controlled by D9D/SCD1 [37]. Whilst D9D/SCD1 is positively correlated with PPARγ to increase lipogenesis in adipocytes [20,37], the significance of reducing the D9D/SCD1 expression to reverse obesity has been constantly discussed [38,39]. Many anti-obesity compounds are designed with the aim of reducing the level of D9D/SCD1 [40,41], similar to the effect shown here by EPA. Bigger LDs has been shown to strongly correlate with the upregulation of PPARγ, D9D/SCD1 and Cidea in adipocytes supplemented with STA and OLA. As illustrated in Figure 5, it is clear that the anti-adipogenic effect of EPA is regulated by the reduction in PPARγ level, which decreased D9D/SCD1 and Cidea gene expressions, followed by a decline in lipid accumulation to induce smaller LDs. However, it is not known whether D9D/SCD1 and Cidea are independent of each other.

It is plausible that modifications in lipolyic pathways influence LD formation and size regulation. LPL and HSL are the major rate-limiting lipases for a balanced lipolysis and lipogenesis in adipocytes. In this study the degree of unsaturation of FAs did not alter the mRNA levels of LPL and HSL in adipocytes. Both EPA and STA (and OLA to some extent) affect the expressions of these lipases at the same magnitude in adipocytes. This results support previous studies on the influence of FAs on lipolytic enzymes gene expressions. Dietary fish oil supplementation increased LPL gene expression in adipose tissue of humans and rats [42,43]. In comparison to other FAs, n-3 PUFA-rich perilla oil did not change LPL expression in rat adipose tissue although a reduced adipogenesis was observed [44]. Similarly, HSL level is only slightly affected by the degree of FA unsaturation, confirming a previous notion [45]. It is speculated that like other FAs, EPA may play a role in lipolytic regulation, however this does not influence the biogenesis of LDs in adipocytes.

Adipocytes produce numerous amounts of inflammatory adipokines upon stimulation. Hypertrophied adipocytes are insulin resistant, and demonstrate greater secretion of low grade inflammatory cytokines, chemokines and vascular proteins [46]. Our study demonstrated that adipocytes differentiated with EPA suppressed gene expressions of LPS-induced pro-inflammatory adipokines, MCP-1 and IL-6. Alternatively, similar treatment resulted in elevated adiponectin and leptin gene expressions compared with SFA, complementing data described elsewhere [47,48]. Thus, EPA-treated adipocytes, with smaller LDs, have improved inflammatory response.

## Conclusion

EPA, being the predominant long chain n-3 PUFA found in marine oils, suppresses LD formation and size in 3T3-L1 adipocytes, in comparison to other FAs. Several lines of evidence suggest a beneficial impact of EPA on lipid accumulation and body weight regulation [21,23,49]. Suppression of LD formation and size may be one novel contributing factor. In addition to examining LD size, we explored the gene analysis of known regulators of LD function. Interestingly, addition of EPA during adipocyte differentiation decreased PPARγ, Cidea and D9D/SCD1 without affecting GLUT4 and Perilipin A expressions, as compared with SFA. Such effects concomitantly suppressed the total lipid accumulation and biogenesis of LDs. Complementing this data is altered expression of adipokines following acute LPS exposure, with EPA-treated adipocytes expressing improved inflammatory response. Thus, EPA is a novel regulator of LD formation, a key intracellular organelle central to the function and regulation of adipocyte metabolism.

## Methods

### Reagents

Dexamethasone (Dex; D4902), isobutylmethylxanthine (IBMX; I5879), insulin (I5500), low-endotoxin FA-free BSA (A8806), LPS (L6529), EPA (44864), STA (85679) and OLA (O1383) (all FAs were with purity > 99%) were obtained from Sigma (St. Louis, MO). Fetal bovine serum (FBS) and DMEM were purchased from Invitrogen (Carlsbad, CA). FAs were dissolved in 100% ethanol as stock solutions of 100 mM while LPS was dissolved in dH_2_O at 1 mg/mL stock. All stock solutions were stored in the dark at -20°C before use. All other reagents were of analytical grade.

### Cell culture

The 3T3-L1 mouse embryo fibroblasts were obtained from the American Type Culture Collection (Rockville, MD). The adipocyte cell culture and their differentiation from 3T3-L1 pre-adipocytes were carried out as described previously [50]. Briefly, cells were seeded in 6-, 12- and 24-well tissue culture plates and maintained in growth medium containing DMEM supplemented with 10% FBS, 2 mM/L glutamine, 100 U/L penicillin and 100 μg/mL streptomycin in a humidified atmosphere of 95% air/5% CO_2 _at 37°C. At post-confluent, adipogenesis of 3T3-L1 was induced with growth media containing 520 μM IBMX, 200 nM Dex and 2 μg/mL insulin. After 3 days of exposure to the differentiation medium, cells were maintained in growth medium containing insulin alone. FA treatment was introduced throughout all 7 days of cell differentiation at physiological concentration of 100 μM. STA was diluted in warm growth medium containing 2% BSA and incubated for 4 hours at 37°C until dissolved. All other FAs were freshly prepared from the stock solution and diluted with growth medium containing 2% BSA. A corresponding amount of 2% BSA + 100% ethanol was used as the vehicle control (CTRL). For LPS stimulation, mature adipocytes were serum starved for 2 h prior to treatment with 1 μg/mL of LPS for 60 min at 37°C to induce inflammation.

### Oil Red O staining

After appropriate treatment, adipocytes were washed with cold phosphate-buffered saline (PBS; pH 7.4) and fixed with 4% paraformaldehyde solution in PBS. Cells were stained with freshly prepared Oil Red O dye (0.5% (w/v) dissolved in isopropanol and diluted at 3:2 ratio of dye:water). Cells were washed thoroughly with distilled water prior to quantification. Spectrophotometric analysis of the stain was performed by dissolving the stained LDs with isopropanol and measuring at A_520 nm _[51]. The values were calculated as percentage of the vehicle control and expressed as means ± SEM (n = 6).

### RNA isolation and gene expression quantification

Total RNA was extracted from 3T3-L1 adipocytes using Tri-reagent (PE Applied Biosystems, Foster City, CA). The purity of the RNA was measured at A_260 nm_/A_280 nm _using Nanodrop 1000 (Thermo Fisher Scientific Inc, MA, USA) with ratio values of ~2.0. The cDNAs was synthesised from 2 μg of RNA using high capacity RNA-to-cDNA reverse transcriptase kit in a total of 20 μL of reaction volume (PE Applied Biosystems). Reverse transcription was performed with sample incubation at 42°C for 1 h, followed by 99°C for 5 min and 4°C for another 5 min. RT-PCR amplifications were performed from 1 μL cDNA diluted at 1:20 using each of the gene-specific primer sets (GeneWorks Pty Ltd, SA, Australia). The oligonucleotide sequence of the forward (sense) and reverse (antisense) primers used for amplification were as in Table 2. Each primer set was used at a concentration of 200 μM in a final volume of 14 μL using Power SYBR^® ^Green PCR Master Mix (PE Applied Biosystems). All PCR reactions were performed using a fluorometric thermal cycler (7500 Fast Real-Time PCR System, PE Applied Biosystems). The ΔΔC_T _method was used to measure relative quantification, where values were normalised to the reference gene (cyclophilin). Individual C_T _values are means of triplicate measurements, with repeatability measurement of < 0.5. Separate control experiments were used to demonstrate that the efficiency of target and reference amplifications were equal.

**Table 2:** Gene specific forward and reverse primer sequences used for RT-PCR

Gene	Forward primer (5' - 3')	Reverse primer (5' - 3')	NCBI accession number
Adiponectin	AAGGACAAGGCCGTTCTCT	TATGGGTAGTTGCAGTCAGTTGG	NM_009605
Cidea	TGCTCTTCTGTATCGCCCAGT	GCCGTGTTAAGGAATCTGCTG	NM_007702
Cyclophilin	TCTGCTGTCTTTGGAACTTTGTC	CTGATGGCGAGCCCTTG	NM_008907
D9D/SCD1	ATGCCGGCCCACATGCTCCAA	TCAGCTACTCTTGTGACTCCC	NM_009127
GLUT4	TGTGGCCTTCTTTGAGATTGG	CTGAAGAGCTCGGCCCACAA	NM_009204
HSL	CCTACTGCTGGGCTGTCAA	CCATCTCGCACCCTCACT	NM_010719
IL-6	ACAAGTCGGAGGCTTAATTAGACAT	TTGCCATTGCACAACTCTATTC	NM_031168
Leptin	TCCAGAAAGTCCAGGATGACAC	CACATTTTGGGAAGGCAGG	NM_008493
LPL	AGTAGACTGGTTGTATCGGG	AGCGTCATCAGGAGAAAGG	NM_008509
MCP-1	CTTCCTCCACCACCATGCA	CCAGCCGGCAACTGTGA	NM_011333
Perilipin A	TGCTGGATGGAGACCTC	ACCGGCTCCATGCTCCA	NM_001113471
PPARγ	GGAATGGGAGTGGTCATCCA	CCCACCAACTTCGGAATC	NM_001127330

### Statistical analysis

All results are expressed as means ± SEM. The statistical significance of differences among groups was determined by one-way analysis of variance (ANOVA) using the SPSS package program version 17.0 (SPSS, Chicago, IL). The results were considered to be significant if the value of *P *was < 0.05 using Tukey's post hoc test.

## Abbreviations

**ATGL**: adipose triglyceride lipase or desnutrin or Pnpla2; **Cidea**: cell death-inducing DNA fragmentation factor alpha subunit-like effector A; **D9D/SCD1**: delta-9 desaturase/stearoyl Co-A desaturase 1; **Dex**: dexamethasone; **EPA**: eicosapentaenoic acid (C20:5n-3); **FA**: fatty acid; **GLUT4**: glucose transporter type 4; **HSL**: hormone sensitive lipase or *Lipe*; **IBMX**: isobutylmethylxanthine; **IL-6**: interleukin-6; **LD**: lipid droplet; **LPL**: lipoprotein lipase; **LPS**: lipopolysaccharide; **MCP-1**: monocyte chemoattractant protein-1; **OLA**: oleic acid (C18:1n-9); **PPARγ**: peroxisome proliferator-activated receptor gamma; **SFA**: saturated fatty acids; **STA**: stearic acid (C18:0); **TAG**: triacylglycerols or triglycerides

## Competing interests

The authors declare that they have no competing interests.

## Authors' contributions

EM conceived the study, its design and coordination, performed all experiments and statistical analysis, discussion of results and drafted the manuscript. AJS participated in the design and coordination of the study and discussion of results. DCS participated in the design and coordination of the study, discussion of results and providing additional funding for the study. All authors read and approved the final manuscript.
